# Sequential autoencoders for feature engineering and pretraining in major depressive disorder risk prediction

**DOI:** 10.1093/jamiaopen/ooad086

**Published:** 2023-10-09

**Authors:** Barrett W Jones, Warren D Taylor, Colin G Walsh

**Affiliations:** Department of Biomedical Informatics, Vanderbilt University Medical Center, Nashville, TN, United States; Department of Psychiatry and Behavioral Sciences, Vanderbilt University Medical Center, Nashville, TN, United States; Geriatric Research, Education, and Clinical Center, Veterans Affairs Tennessee Valley Health System, Nashville, TN, United States; Department of Biomedical Informatics, Vanderbilt University Medical Center, Nashville, TN, United States; Department of Psychiatry and Behavioral Sciences, Vanderbilt University Medical Center, Nashville, TN, United States; Department of Medicine, Vanderbilt University, Nashville, TN, USA

**Keywords:** deep learning, depressive disorder, major, longitudinal studies, supervised machine learning, electronic health record

## Abstract

**Objectives:**

We evaluated autoencoders as a feature engineering and pretraining technique to improve major depressive disorder (MDD) prognostic risk prediction. Autoencoders can represent temporal feature relationships not identified by aggregate features. The predictive performance of autoencoders of multiple sequential structures was evaluated as feature engineering and pretraining strategies on an array of prediction tasks and compared to a restricted Boltzmann machine (RBM) and random forests as a benchmark.

**Materials and Methods:**

We study MDD patients from Vanderbilt University Medical Center. Autoencoder models with Attention and long-short-term memory (LSTM) layers were trained to create latent representations of the input data. Predictive performance was evaluated temporally by fitting random forest models to predict future outcomes with engineered features as input and using autoencoder weights to initialize neural network layers. We evaluated area under the precision-recall curve (AUPRC) trends and variation over the study population’s treatment course.

**Results:**

The pretrained LSTM model improved predictive performance over pretrained Attention models and benchmarks in 3 of 4 outcomes including self-harm/suicide attempt (AUPRCs, LSTM pretrained = 0.012, Attention pretrained = 0.010, RBM = 0.009, random forest = 0.005). The use of autoencoders for feature engineering had varied results, with benchmarks outperforming LSTM and Attention encodings on the self-harm/suicide attempt outcome (AUPRCs, LSTM encodings = 0.003, Attention encodings = 0.004, RBM = 0.009, random forest = 0.005).

**Discussion:**

Improvement in prediction resulting from pretraining has the potential for increased clinical impact of MDD risk models. We did not find evidence that the use of temporal feature encodings was additive to predictive performance in the study population. This suggests that predictive information retained by model weights may be lost during encoding. LSTM pretrained model predictive performance is shown to be clinically useful and improves over state-of-the-art predictors in the MDD phenotype. LSTM model performance warrants consideration of use in future related studies.

**Conclusion:**

LSTM models with pretrained weights from autoencoders were able to outperform the benchmark and a pretrained Attention model. Future researchers developing risk models in MDD may benefit from the use of LSTM autoencoder pretrained weights.

## Background and significance

The use of electronic health record (EHR) data for clinical risk modeling has become increasingly prevalent in recent years.^1^ However, EHR data are high dimensional and complex, posing significant challenges for effective analysis. Some of the complexity inherent in EHR data includes temporality, noise, and sparsity, which can negatively impact predictive performance.^1–3^ To address these challenges, autoencoder models have emerged as a promising approach for generating simplified representations that reduce dimensionality, denoise, and account for temporality.^4–6^ Moreover, pretrained weights can reduce training time and increase predictive performance.^7–9^ Previous studies have shown that autoencoders and other pretrained encoding models can achieve state-of-the-art prediction accuracy in diagnostic tasks and may learn complex disease relationships.^1^^,^^10^

These approaches may be particularly important for common psychiatric disorders, including major depressive disorder (MDD). Machine learning applications have been widely used for prognostic prediction to support clinicians in the identification of individuals with MDD at elevated risk for suicidal behavior.^11–17^ Creating models with clinical benefits in this syndromal phenotype is particularly difficult. In a recent meta-analysis,^13^ a majority of risk models considered had a precision of less than 1%, resulting in concerns about the clinical usefulness of suicidality risk models and a negative relationship between model performance and study quality may exist.^15^ It has been shown that for cost-effectiveness suicide-attempt models should exceed a precision of 0.8%.^18^ Low predictive performance in these studies can in part be explained by class imbalance in training datasets, and lack of clear evidence for suicidality risk factors.^19^ These studies present an opportunity for innovative machine learning techniques to improve predictive performance and clinical benefit of risk models in the MDD population.

It is common practice for researchers working with EHR data to generate aggregate features for prediction of outcomes relevant to MDD. Due to the sparsity of outcomes and features in the patient population, autoencoders have potential to improve predictive performance. Tran et al.^20^ show that restricted Boltzmann machine (RBM) encodings improved prediction in patients under suicide risk assessment. A recent review of deep learning techniques for automated feature representation identified 49 recent publications in which automated feature representation was applied to a range of prediction tasks.^1^ Autoencoder pretraining has been shown to improve predictive performance in biomedical prediction tasks.^8^^,^^9^ Autoencoder feature engineering has also been applied to patient subtyping,^1^ treatment trajectory characterization,^6^ and causal inference.^21–23^

Autoencoders are composed of encoder and decoder submodels that have the capability to represent complex feature dependencies.^4^ The encoder model, $f_{\theta }$, maps the inputs, x, to a lower-dimensional latent space representation, $z=f_{\theta }(x)$. The decoder model, $g_{\theta^{\prime }},$ maps this latent vector back to the original input space to reconstruct the input $x^{\prime }=g_{\theta^{\prime }}(z)$. The model is fit by minimizing the error between the original input and reconstructed inputs. Autoencoders can flexibly accommodate various encoder and decoder model architectures. To account for the temporal nature of EHR data, this work focuses on the use of sequential neural networks.

## Objective

The objective of this study was to evaluate autoencoder ability to improve predictive performance through feature engineering and pretraining across multiple prediction tasks in MDD. We evaluated the autoencoder model’s ability to capture temporal disease relationships that may not be identified by aggregate features and whether that information is retained in the model encodings, z, or the pretrained weight values $f_{\theta }$. The predictive performance of autoencoder models of multiple structures were investigated in an array of clinical outcomes, including unplanned admissions, emergency department (ED) visits, high utilization, and self-harm/suicide attempt. The included health utilization outcomes may ease the challenges of suicidality risk prediction and maintain clinical relevance—through association with MDD severity.^24–26^ To evaluate autoencoders as a feature engineering technique, encodings are input to a random forest model for prognostic prediction, as random forests have shown strong prediction performance in the MDD population in prior studies.^12^^,^^17^ To evaluate autoencoder pretraining, encoder weights are extracted from the autoencoder models and used to initialize neural network prediction models. Predictive performance for autoencoder feature engineering were compared to benchmarks of a random forest trained on aggregate features and an RBM as in Tran et al.^20^ Pretraining predictive performance is compared between long-short-term memory (LSTM) and Attention neural network models of the same structure, but without pretraining, as well as the best performing feature engineering technique.

## Methods

### Data description

This study examined data from the Vanderbilt University Medical Center (VUMC) Research Derivative.^27^ VUMC is located in the United States mid-south, Nashville, Tennessee. The Research Derivative includes data from multiple clinical systems that are structured for research purposes. Patients were included in the study having an MDD indication between January 1, 2013 and December 31, 2018. We defined MDD indication as a depression-related International Classification of Diseases (ICD) diagnosis code, including MDD, dysthymic disorder, and depressive disorder not elsewhere classified,^28^^,^^29^ an antidepressant prescription, or problem list mention of depression. The ICD codes are in supplementary materialsTable S1 and the list of antidepressants Table S2. We additionally required that patients had a depression-related ICD code during the time period of analysis, are 18-90 years old at indication, had 2 visits 6 months apart at VUMC prior to indication, and were not diagnosed with bipolar disorder or schizophrenia. Study data were extracted from the research derivative using IBM Netezza SQL and preprocessing was done in Python (version 3.8).

For patients meeting entry criteria, we extracted 3 years of data after the initial MDD indication and formatted it into a quarterly time series with feature indicators. Feature categories included diagnoses, interventions, and outcomes. ICD diagnosis codes^28^ were extracted and grouped using Agency for Healthcare Research and Quality Clinical Classification Software (CCS)—a grouping of ICD codes intended to be clinically meaningful.^30^

Study interventions were identified and defined with clinical expert guidance (WDT) and were extracted from orders and notes data. Interventions included prescribing one or multiple antidepressants, antidepressant dose change, psychotherapy referral, partial hospitalization referral, and electroconvulsive therapy (ECT) referral. Interventions that were not related to medications were supplemented by notes data. Regular expressions were developed to identify mentions of a referral or consult for psychotherapy, ECT consultation, and partial hospitalizations in both the notes and order data. Antidepressant data were extracted by Anatomic Therapeutic Chemical (ATC) code^31^ according to a list of antidepressants identified by collaborating psychiatrists. The list of antidepressants has been included in supplementary materialsTable S2 with ATC code.

Outcomes included self-harm/suicide attempts, unplanned admission, ED visits, and high utilization. Unplanned admissions were defined as patients admitted to the hospital excluding any admissions that may be considered part of planned treatment according to the Center for Medicare and Medicaid Services Unplanned Readmissions Algorithm.^32^ High utilization was defined as any patient with two or more inpatient or emergency room visits with an MDD-related ICD code during a quarter. The self-harm/suicide attempt outcome is ICD code based, where ICD codes were mapped to the self-harm/suicide attempt CCS code.

### Autoencoder architectures

Autoencoders are composed of two sub-models—an encoder and decoder. The encoder takes the input data and outputs a latent representation. The latent representation is input to the decoder model from which it reconstructs the input data. We test neural network architectures that account for the sequential nature of time series data. LSTMs are a form of recurrent neural network (RNN) that stores information over extended sequences and employs a gating method to address the exploding gradient problem found in some RNN applications.^33^ Attention-based architectures learn long-term dependencies and have been shown to outperform LSTMs on natural language processing tasks.^34^ In contrast to RNNs that keep a state representation that is updated at each position of the sequence, the attention mechanism identifies valuable past information given the current state.

**Figure 1.**
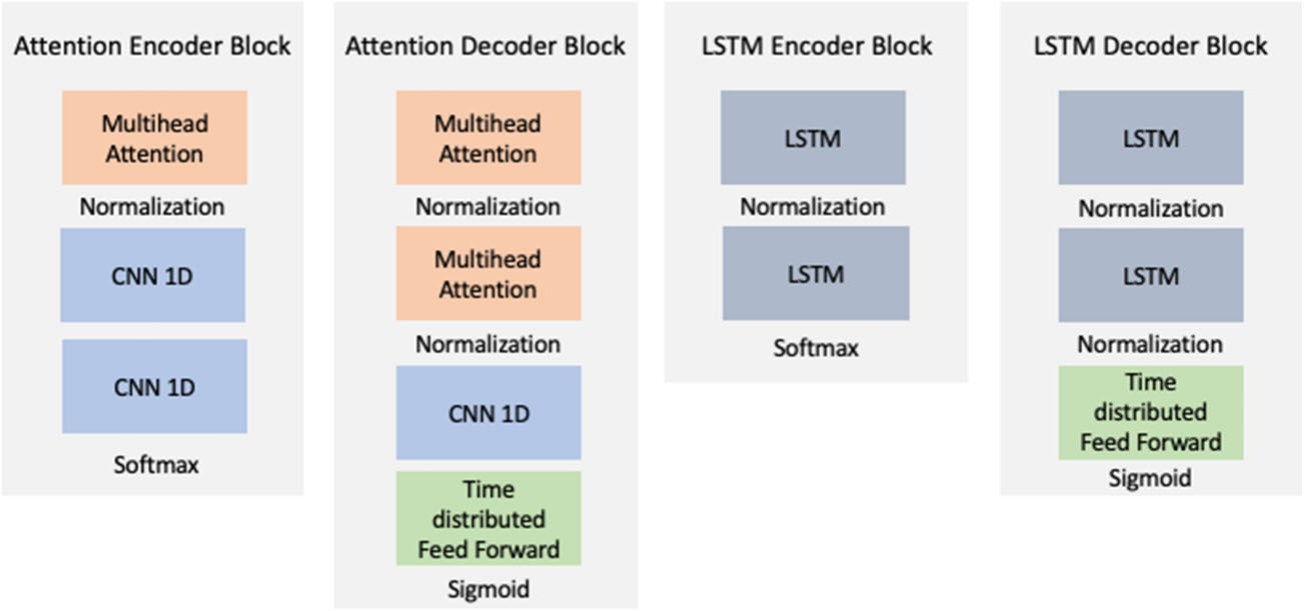
Attention and LSTM model encoder and decoder blocks. Attention encoder block has a single multihead attention layer followed by 2 single dimension convolutional neural network (CNN) layers. The attention decoder has 2 multihead attention layers followed by a CNN and time distributed feedforward layer. The sigmoid activation on the final layer outputs probability estimates of the input indicators. The LSTM encoder block is composed of 2 LSTM layers and the decoder 2 LSTM layers followed by a time-distributed feed-forward layer that estimates the probabilities of the inputs.

The attention model architecture was adapted from Vaswani et al. to work with time series features.^34^ The Keras (version 2.12.0) python library was used to construct the autoencoder models.^35^ The Attention encoder block is composed of a multihead attention layer followed by 2 1-dimensional convolutional neural network (CNN) layers. The decoder block is composed of 2 multihead attention layers followed by a 1-dimensional CNN layer and a time-distributed feed-forward layer. The multihead attention layer computes multiple self-attention layers in parallel to attend to different parts of the input sequence simultaneously. The CNN layer learns a convolution kernel across the time series vector and is able to capture local patterns in the sequence.^36^ The time distributed feedforward layer applies a fully connected layer to each timestep in the sequence. This layer has a sigmoid activation to estimate the probability of the original inputs. The LSTM encoder includes two LSTM layers in both the encoder and decoder blocks and time-distributed feed-forward layer in the decoder block. Figure 1 displays the structures of the attention and LSTM encoder and decoder blocks. Further details on layer parameters are included in supplementary materialsTable S3.

### Benchmark models

As a benchmark feature representation, the time series were aggregated by yearly rolling feature counts at each timepoint. A random forest model was fit with grid search cross validation of hyperparameters for each study outcome. RBM’s have been shown to achieve state-of-the-art predictive performance in the MDD phenotype.^20^ We evaluated an augmented version of the model of Tran et al. optimized for the prediction tasks of this study. Time series data were aggregated indicator features corresponding to (0-90), (90-180), (180-360), and (360-720) day intervals. An RBM with elasticnet regression pipeline was fit with concurrent hyperparameter tuning to optimize AUPRC. RBM-tuned parameters included a number of components and learning rate. LSTM and Attention neural networks were composed of the LSTM and Attention encoding blocks (see Figure 1), without pretraining, followed by a time-distributed dense layer. An LSTM and Attention model were fit for each outcome.

### Model training and evaluation

Patients meeting entry criteria were split, two-thirds into a training and one-third into a test set. The quality of each representation is evaluated in each study outcome. Autoencoder models were fit on the training data and at each time point, we fit a random forest model with grid search hyper parameter tuning with autoencoder latent vectors as input. Weights were extracted from the encoders of both the LSTM and Attention autoencoders. A time-distributed dense layer was appended to the encoding layers and a predictive model was trained on each of the study outcomes. The area under the precision-recall curve (AUPRC) was recorded for each predictive model on the test set. We recorded AUPRC at each time point on the test set, and trends and variations were evaluated.

## Results

Of the 27 319 patients meeting entry criteria 17 621 (64.5%) were female. Most patients are non-Hispanic/Latinx whites (*n* = 22 478, 82.3%). Black patients account for 11.0% of the population, with 1027 (3.8%) of patients being classified as other, this includes patients with unreported or multiple reported races. The most common intervention in the study is prescribing an antidepressant (*n* = 19 414, 71.1%), of those 42% are prescribed multiple. Table 1 contains details on the patient population, count data are aggregated across the entire study period.

**Table 1.** Patient descriptive statistics aggregated across the entire study period.

	*N*	%
Gender		
Female	17 621	64.5%
Race/ethnicity		
American Indian or Alaska Native	52	0.2%
Asian	379	1.4%
Black	2996	11.0%
White-Hispanic/Latinx	387	1.4%
White-not Hispanic/Latinx	22 478	82.3%
Other	1027	3.8%

	**Mean**	**SD**

Age	48.1	18.1

**Outcomes**	***N***	%

Unplanned admission	11 172	40.9%
ED visit	5278	19.3%
High utilization	968	3.5%
Self-harm/suicide attempt	2032	7.4%
Interventions		
Antidepressant prescription	19 414	71.1%
Multiple antidepressant prescriptions	8300	30.4%
Dose increase	1838	6.7%
Dose decrease	922	3.4%
Psychotherapy referral	7592	27.8%
ECT referral/consult	101	0.4%
Partial hospitalization referral/consult	213	0.8%

**Diagnoses**	**Mean**	**SD**

CCS code count (unique)	14.3	10.3

**Figure 2.**
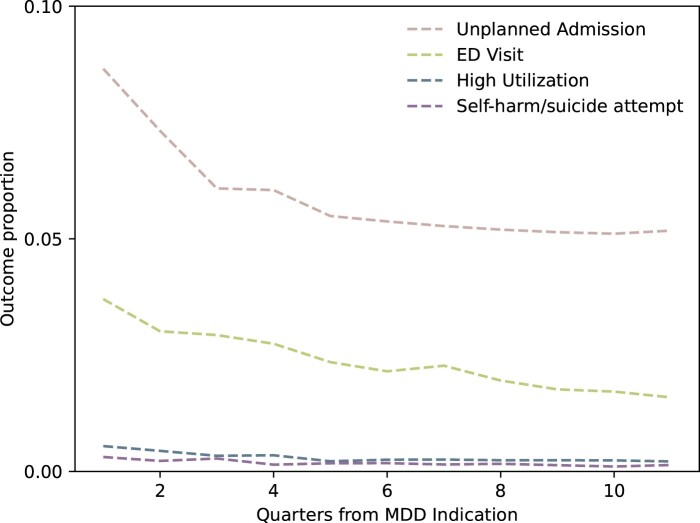
Outcome frequency trend by quarter. The proportion of patients with the observed outcomes is plotted at each timepoint in the study.

Through the entire study period 11 172 (40.9%), patients have an unplanned admission, by quarter this outcome ranges in proportion from 5.1% to 8.7%. ED visit proportions by quarter range from 1.5% to 3.6%. Each of the study outcomes had a decreasing trend across the study period (ordinary least square *P*-value <.001). High utilization and self-harm/suicide attempt are relatively infrequent, ranging from 0.20%-0.54% to 0.13%-0.31% respectively. Figure 2 shows outcome proportion trends by quarter.

The training set included 18 213 patients. Autoencoders were trained with a 10% validation set and binary cross-entropy loss. The final validation error was 0.012 for the Attention autoencoder with 997 314 trainable parameters. The validation loss for the LSTM autoencoder was 0.006 with 717 066 trainable parameters.

**Figure 3.**
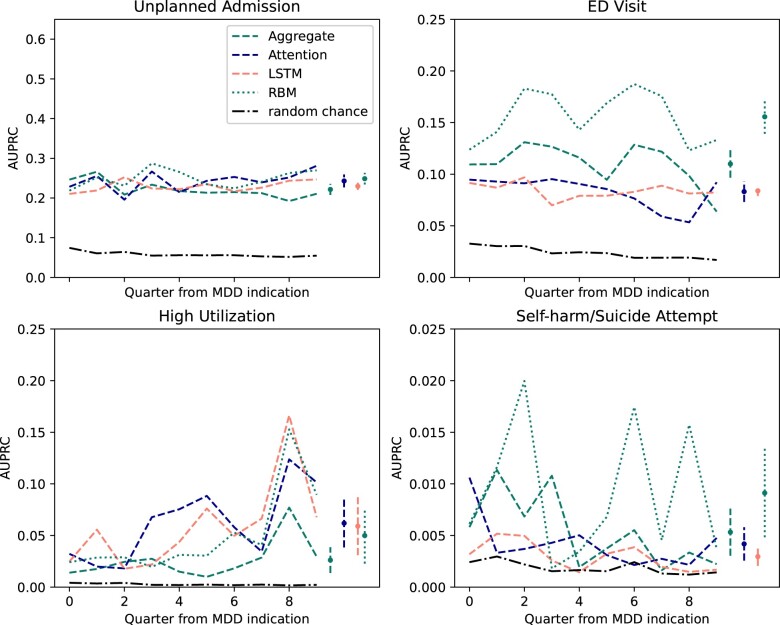
Temporal validation of feature engineering methods. Each line displays the AUPRC trend for the corresponding feature engineering method. Plots are included for temporal validation of feature engineered training data at each quarter in the study. The black dot-dash line represents the performance of a random chance estimator. 95% confidence intervals are shown for the average AUPRC across the study time period.

The predictive performance of each autoencoder as a feature engineering method was evaluated temporally by fitting a random forest on model encodings with grid search cross-validation parameter tuning. Test AUPRC scores were calculated at each time point and are reported in Figure 3. RBM had the highest average AUPRC across each prediction task, except for high utilization, where the LSTM and Attention feature engineering had higher AUPRC (RBM 0.050, 95% CI 0.023-0.077, LSTM 0.059, 95% CI 0.031-0.087, Attention 0.062, 95% CI 0.038-0.085). RBM had significantly better AUPRC for the ED visit outcome (0.16, 95% CI 0.14-0.17), where next best was aggregate feature engineering (0.11, 95% CI 0.10-0.12). Relatively high variance in AUPRC was observed in the self-harm/suicide attempt outcome—RBM ranges from 0.0018 to 0.020. LSTM compared to Attention-based autoencoder feature engineering was similar across outcomes. Attention had higher average AUPRC in the unplanned admission, high utilization, and self-harm/suicide attempt outcomes.

**Figure 4.**
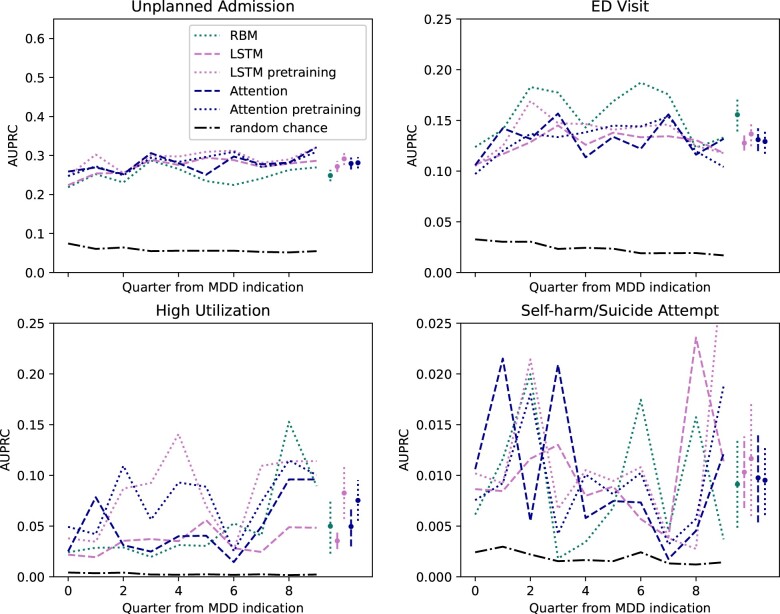
Temporal validation of pretraining methods. LSTM and Attention pretrained models were compared to the same model structure without pretraining. The RBM model was best performing of feature engineering methods and is included in this graphic. The black dot-dash line represents the performance of a random chance estimator. 95% confidence intervals are shown for the average AUPRC across the study time period.

The LSTM model with pretraining had the highest average AUPRC in 3 of 4 outcomes (Figure 4). The exception is the ED visit performance where RBM has the highest AUPRC (RBM = 0.16, 95% CI 0.14-0.17, LSTM pretrained = 0.14, 95% CI 0.12-0.15). Pretraining resulted in an increase in performance over LSTM without pretraining in each outcome. LSTM with pretraining had highest average AUPRC in the self-harm/suicide attempt outcome, but due to variation over time, the result is not a significant improvement over benchmark. Pretrained attention models had comparable performance relative to attention without pretraining. Attention without pretraining had a higher average AUPRC in ED visits, and self-harm/suicide attempts. The self-harm/suicide attempt LSTM pretrained model had a precision of 1.45%, recall of 30.08%, and specificity of 95.31% for the top 5% of risk predictions in patients with observed features during the prediction quarter.^37^

## Discussion

This study evaluated autoencoder feature engineering and pretraining in MDD patients across an array of prediction tasks. Autoencoder models selected for this study account for temporality, denoise, reduce dimensionality, and capture interactions between EHR features. When using pretrained weights, we were able to improve predictive performance over benchmarks in 3 of 4 outcomes. The pretrained weights improved predictive performance in the LSTM, relative to a model with the same architecture and no pretraining. This suggests that pretrained information from the autoencoders may be best retained in the model weights. In contrast, encodings as input to a random forest model did not improve predictive performance. Improvement in prediction resulting from pretraining has potential for increased clinical usefulness of risk models in MDD and other clinical areas, with a test precision in the self-harm/suicide attempt outcome over 1%.^13^^,^^18^^,^^37^

Feature encodings from LSTM and Attention autoencoders were not superior in predictive performance relative to aggregate feature engineering. Random forest models have a decision tree structure that accounts for feature interactions and ensembles decision trees to protect from overfitting to noise in the training data.^38^ It appears that the random forest model’s structure was sufficient to partially account for noise and complex interactions while the benefit of temporality captured in encodings in the LSTM and Attention varied across prediction tasks. We observed information loss in training each autoencoder format, as none were able to achieve zero validation loss. The information lost in encoding estimation may contribute to the lack of performance of autoencoder feature engineering, while this information may have been retained in autoencoder weights.

Encodings from the RBM model resulted in best AUPRC for all but one outcome of the feature engineering techniques. The RBM modeling strategy used has been shown to outperform principal component analysis as a feature engineering technique in a suicide risk prediction task and has the capability to learn complex interactions between high dimensional features. Additionally, the RBM has a low number of trainable parameters (29 747-74 147) relative to the LSTM and Attention autoencoders, suggesting LSTM and Attention autoencoders could be overfit.

We observed variation in performance between autoencoder configurations with the LSTM pretraining model having the most consistent performance across prediction tasks. Attention-based models have outperformed LSTMs in many sequential data prediction tasks, specifically in natural language processing.^10^^,^^34^^,^^39^^,^^40^ Attention models are effective in part because of their ability to efficiently learn long-range dependencies in a sequence. However, in our study, the sequences are relatively short compared to NLP applications where attention-based models have been superior. Additionally, the structure of attention models allows for increased parallelization—speeding up training relative to LSTMs. In our study, the model trainable parameters and number of training examples were such that training time was relatively short. The nature of this study may nullify the advantages attention models have over LSTMs in other studies.

Average AUPRCs were low for the self-harm/suicide attempt outcome across prediction techniques. Self-harm/suicide attempt events were relatively rare compared to the other outcomes, except for high utilization. The high utilization outcome has a similar frequency, but pretrained LSTM has a mean AUPRC more than 7 times that of self-harm/suicide attempt. Since EHR data reflect healthcare utilization, EHR-based features may provide higher prediction performance in utilization-based outcomes. Overall, healthcare utilization is common in patients prior to suicide events, although not always mental healthcare-specific utilization.^41^ Further study of health utilization events that precede suicidality on the causal pathway could allow for the training of prediction models with increased clinical precision.

This study highlights several implications for the use of autoencoders for prediction tasks in the MDD population. Evidence of the benefits of autoencoder pretraining is shown with a limited dataset at a single site. Researchers considering the development of predictive models in this patient population may improve predictive performance with this training strategy. It is possible the benefits of autoencoder pretraining will extend to additional clinical areas.^8^ LSTM performance relative to Attention architectures suggests that LSTM architectures should also be considered when working with similar datasets. Observed autoencoder information loss, specifically in Attention architectures, could have been due to the lack of training examples. Future studies of multisite data may have better performance in Attention architectures and allow for additional training techniques such as self-supervised learning.^10^ We observed higher AUPRCs in health utilization outcomes relative to self-harm and suicide attempts. Researchers in this space should consider the actionability and clinical usefulness of these or related health utilization outcomes when developing risk models for MDD patients.

This study is limited to a single site and single mental health phenotype. It is possible that the study results will not generalize to other phenotypes and healthcare systems. We study multiple autoencoder structures of varying model sizes. However, there are many alternative structures not studied here that may result in improved performance.

## Conclusion

We evaluate temporal autoencoder pretraining and feature engineering in the MDD population and compare predictive performance to a benchmark modeling strategies that have proven successful in the MDD phenotype.^17^^,^^20^ LSTM models with pretrained weights from autoencoders were able to outperform the benchmark, as well as an equivalent LSTM model without pretraining. Autoencoder feature engineering was unable to outperform the benchmark. This suggests that information retained by model weights may not be passed to encodings. Future researchers developing risk models in MDD may benefit from the use of autoencoder pretrained weights.

## Supplementary Material

ooad086_Supplementary_Data

## Data Availability

The data underlying this article cannot be shared publicly due to privacy of patient medical records analyzed in this study. The data will be shared on reasonable request to the corresponding author.
