# Supplementary material for: Sequential autoencoders for feature engineering and pretraining in major depressive disorder risk prediction
Source: JAMIA Open. 2023 Oct 9;6(4):ooad086. doi: 10.1093/jamiaopen/ooad086 (PMC10561992; doi:10.1093/jamiaopen/ooad086)
Supplement: ooad086_Supplementary_Data [file ooad086_supplementary_data.docx]

**APPENDIX**

Table S1-Depression ICD codes list

|  | Code | Description |
| --- | --- | --- |
| ICD-9 | 311.x | Depressive disorder, not elsewhere classified |
|  | 296.2x | Major depressive disorder single episode |
|  | 296.3x | Major depressive disorder recurrent episode |
|  | 300.4x | Dysthymic disorder |
| ICD-10 | F32.xx | Major depressive disorder, single episode |
|  | F33.xx | Major depressive disorder, recurrent |
|  | F34.1 | Dysthymic disorder |

Table S2-Antidepressant List with ATC code

| Code | Name | Code | Name | Code | Name |
| --- | --- | --- | --- | --- | --- |
| N06BA04 | methylphenidate | N06AA10 | nortriptyline | N06AX16 | venlafaxine |
| N06BA02 | dexamfetamine | N06AA11 | protriptyline | N06AX17 | milnacipran |
| N05AH04 | quetiapine | N06AA04 | clomipramine | N06AX06 | nefazodone |
| N05AH03 | olanzapine | N06AA21 | maprotiline | N06AX23 | desvenlafaxine |
| N05AE04 | ziprasidone | N06AA17 | amoxapine | N03AX09 | lamotrigine |
| N05AN01 | lithium | N06AA02 | imipramine | N05AX12 | aripiprazole |
| N06AF04 | tranylcypromine | N06AX24 | vilazodone | N06AB04 | citalopram |
| N06AF03 | phenelzine | N06AX12 | bupropion | N06AB03 | fluoxetine |
| N06AF01 | isocarboxazid | N06AX21 | duloxetine | N06AB05 | paroxetine |
| N06AA12 | doxepin | N06AX26 | vortioxetine | N06AB10 | escitalopram |
| N06AA09 | amitriptyline | N06AX05 | trazodone | N06AB08 | fluvoxamine |
| N06AA01 | desipramine | N06AX11 | mirtazapine | N06AB06 | sertraline |

Table S3 Autoencoder model parameters

| Attention Encoder | Layers |
| --- | --- |
|  | Multi-head-attention(head size = 64, number of heads = 4) |
|  | Convolutional 1D(filters = 294, kernel size = 6, activation = 'relu') |
|  | Convolutional 1D(filters = 147, kernel size = 3, activation = 'relu') |
| Attention Decoder |  |
|  | Multi-head-attention(head size = 64, number of heads = 4) |
|  | Multi-head-attention(head size = 64, number of heads = 4) |
|  | Convolutional 1D(filters = 294, kernel size = 6, activation = 'relu') |
|  | Time distributed dense(units = 147, activation = 'sigmoid') |
|  |  |
| LSTM Encoder |  |
|  | LSTM(units=147, activation='relu') |
|  | LSTM(units=147, activation='relu') |
| LSTM Decoder |  |
|  | LSTM(units=147, activation='relu') |
|  | LSTM(units=147, activation='relu') |
|  | Time distributed dense(units = 147, activation = 'sigmoid') |
